# Evaluating the Influence of Food Trade on Human Exposure to Heavy Metals via Crops in China

**DOI:** 10.3390/toxics14060474

**Published:** 2026-05-28

**Authors:** Bo Tang, Xuhua Miao, Jianyuan Ma, Wenxiu Liu, Qingbao Gu, Fujun Ma

**Affiliations:** 1 State Key Laboratory of Environmental Criteria and Risk Assessment, Chinese Research Academy of Environmental Sciences, Beijing 100012, China; tangbo9703@163.com (B.T.); guqb@craes.org.cn (Q.G.); mafj@craes.org.cn (F.M.); 2 School of Chemical & Environmental Engineering, China University of Mining & Technology (Beijing), Beijing 100083, China; 3 Gansu Academy of Eco-environmental Sciences, Lanzhou 730020, China; mmx22cn@163.com; 4 Gansu Engineering Research Center of Soil Environmental Protection and Pollution Prevention, Lanzhou 730020, China

**Keywords:** HMs, soil pollution, interregional food trade, risk assessment, daily intake

## Abstract

Crops can accumulate heavy metals (HMs) from soil, leading to human exposure through dietary intake. However, the influence of interregional food trade on dietary HMs exposure remains underexplored. In this study, using data derived from existing literature, the occurrence and distribution patterns of six HMs, namely arsenic (As), cadmium (Cd), chromium (Cr), nickel (Ni), lead (Pb), and zinc (Zn), in soil and crops across China were investigated. Furthermore, the influence of food trade on human exposure to soil-derived HMs was assessed. The average total concentration of the six HMs in soil was 234.01 ± 29.54 mg/kg, while concentrations in rice and wheat were 16.06 ± 2.91 mg/kg and 22.48 ± 4.22 mg/kg, respectively. The hazard quotients (HQs) for As in rice exceeded 1 in the Central, Central Coast, South Coast, Southwest, and Northeast regions, indicating potential health risks. Interregional food trade significantly redistributed these risks. Through rice consumption, the Central and Northeast regions accounted for up to 36.78% and 45.08% of the daily intakes of As and Cd in other regions, respectively. Similarly, through wheat consumption, the Central and Southwest regions accounted for up to 51.33% and 25.97% of the daily intakes of As and Cd, respectively. This redistribution is largely attributed to the concentration of major crop production in the Central, Northeast, and Southwest regions. Overall, this study highlights the critical role of interregional food trade in modulating population health risks associated with contaminants, providing a more accurate and comprehensive assessment of dietary HMs exposure.

## 1. Introduction

With the rapid advancement of industrialization and urbanization in China, the issue of heavy metals (HMs) pollution in agricultural soil has garnered significant attention. The presence of these toxic elements in soils is of great concern due to their non-biodegradable nature, resulting in their prolonged persistence within the environment [1]. A previous study documented a notable incidence of soil pollution exceeding regulatory thresholds, with a reported rate of 16.1% [2]. Specifically, concerning HMs, the prevalence of cadmium (Cd), arsenic (As), lead (Pb), and chromium (Cr) surpassing standard limits stands at 7.00%, 2.70%, 1.50%, and 1.10%, respectively. Soil serves as the fundamental substrate for cultivating food crops. Consequently, the consumption of contaminated food crops has emerged as a significant determinant of human exposure to HMs [3], posing risks to the general population [4].

Human activities result in excessive input of HMs into the soil, which can adversely affect the growth of crops. HMs in crops can easily be transferred and accumulated through the food chain, posing a certain threat to human health, such as causing kidney disease and cardiovascular diseases [5,6].

While a considerable number of studies have documented the accumulation of HMs from soil to food, it is noteworthy that these investigations have predominantly focused on assessing the general dietary exposure risks associated with HMs for human populations. However, they have often neglected to specifically estimate the contributions of soil to HMs in assessing human exposure to HMs. In China, it is crucial to acknowledge the significant regional variations that exist within the country. Additionally, the widespread practice of food trade further compounds these regional differences. The process of interregional food trade involves the redistribution of food between regions, which results in alterations in the geographical distribution and exposure risks of contaminants in food [7]. Consequently, such changes have the potential to modify the regional patterns of exposure risks to pollutants [8]. A study showed that methylmercury (MeHg) exposure is increased in regions such as Africa and Europe due to international rice trade, while it is decreased in North America and South America [9]. Similarly, inter-provincial trade within China has been found to exert a significant influence on atmospheric mercury emissions, deposition, and associated risks [10]. Chen et al. investigated the impact of inter-provincial trade on mercury emission and found that the inter-provincial trade could promote the transportation of pollutants between different provinces and reduce the health risks in high-level regions [11,12]. Consequently, this process acts as a preventive measure against mercury-related health risks along the southeast coast but simultaneously leads to additional mercury-related health risks in inland regions. Moreover, the health risks associated with As in the global rice trade were examined, and countries relying solely on imported rice are importing a significant amount of health risk due to As contamination [13,14]. However, there is currently no available study that comprehensively incorporates the impacts of food trade in evaluating the dietary exposure risks of HMs originating from soil contamination in China.

In this study, the spatial distribution of HMs in soil across various regions of China was analyzed, and the concentrations of HMs in rice and wheat were calculated by considering bioconcentration factors (BCFs). Moreover, the regional and national exposure to HMs through wheat and rice consumption was assessed, and the health risks associated with exposure to HMs, taking into account the interregional food trade, were evaluated. By considering the flow of food commodities among different regions, the extent to which HMs were redistributed through food trade could be determined. The objective of this study was to investigate the impact of interregional food trade on the presence of HMs in agricultural soil and to provide a comprehensive evaluation of the risks associated with their contamination in the food supply chain, particularly as related to interregional trade.

## 2. Materials and Methods

### 2.1. The Exposure of HMs in Soil

A comprehensive search was conducted to collect primary research publications reporting concentrations of HMs in agricultural soils across various provinces in China. This search was carried out using databases such as Web of Science (WOS) and China National Knowledge Infrastructure (CNKI). The literature retrieval process was performed using specific keywords, including “heavy metals,” individual elements (such as As, Cd, Cr, Ni, Pb, and Zn), “soil,” “concentration,” and “China.” Based on these criteria, relevant studies providing information on the spatial distribution of HMs in agricultural soils across different provinces were identified. Specifically, concentrations of the six HMs (As, Cd, Cr, Ni, Pb, and Zn) in soils for each province were summarized from the available literature. The original HMs concentration data used in this study are available upon reasonable request after acceptance.

A comprehensive review of peer-reviewed literature was conducted to identify exposure levels of HMs in agricultural soils. More than 50,000 samples from different provinces across China were included in the dataset. To ensure data consistency and representativeness, samples identified as originating from polluted soils were excluded. In this study, “polluted soils” were defined as soils with HMs concentrations exceeding the risk screening values specified in the Chinese Soil Environmental Quality Standard (GB 15618-2018) [15]. When explicit classifications were provided in the original literature, samples reported as contaminated or polluted were excluded accordingly. This screening process was applied to ensure that typical agricultural soil conditions, rather than heavily contaminated sites, were represented in the dataset, thereby avoiding overestimation of dietary exposure risks. After applying these inclusion and exclusion criteria, the compiled dataset covered more than 30 provinces and provided a robust basis for subsequent analysis.

Based on the screened dataset described above, soil HMs concentrations were compiled for more than 30 provinces, covering a population of approximately 1.4 billion in China. HMs concentrations were calculated separately for each province, and the averages were derived to represent soil HMs levels at the provincial scale. To further assess the impact of interregional food trade on HMs concentrations in crops, China was divided into eight regions, as shown in Table 1 [7].

### 2.2. The Measurement of HMs in Wheat and Rice

In this study, the BCFs were utilized to estimate the concentration of HMs in crops (C: mg/kg) considering the transfer from soil, as expressed in the following equation:

(1) C = BCFs × S

Here, S (mg/kg) is the soil HMs level. The BCFs utilized in this study were obtained from previously published literature and summarized in Table 2. More detailed information regarding BCFs of two crops, including rice and wheat, is provided in Table S1.

### 2.3. The Estimated Daily Intake of HMs

Briefly, taking into account the influence of interregional food trade on HMs in crops, the estimated daily intake (EDI) of HMs was determined by employing the following equation [16]:

(2) EDI = C × F

Here, C is the concentration of HMs in crops (mg/kg); F is the food consumption normalized by body weight (g/kg/day) from different regions (details in Supplementary Materials Tables S2 and S3), representing the food consumption of receptor regions from source regions [11]. Unit conversion was applied during the EDI calculation, and the final results are expressed in μg/kg/day.

### 2.4. Human Health Risk Assessment

The HQ method was utilized to assess the potential health risks related to the consumption of HMs via dietary intake. The HQ value was calculated as follows:

(3) $$\mathrm{HQ}\;=\;\frac{EDI}{RfD}$$

Here, RfD is the reference dose for HMs [17,18]. The RfD of As, Cd, Cr, Ni and Zn is 0.30, 1.00, 3.00, 20.00 and 300.00 μg·kg^−1^ day^−1^, respectively (USEPA, 2017) [19]. The RfD of Pb is 1.50 μg·kg^−1^ day^−1^ [20].

## 3. Results and Discussion

### 3.1. National Occurrence of HMs in Agricultural Soil

**Figure 1:**
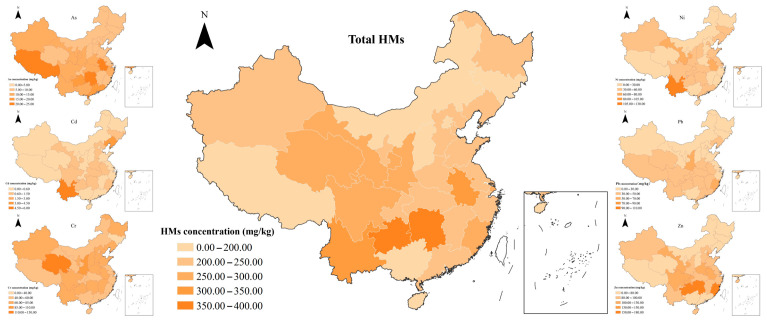
The spatial distributions of different HMs.

**Figure 2:**
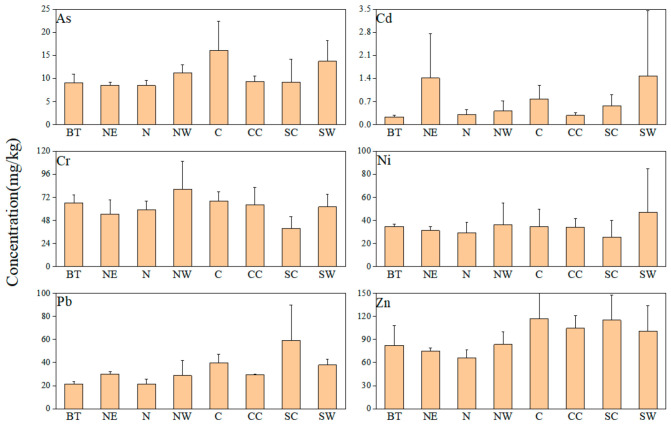
The average sub-regional occurrence of different heavy metals. The eight regions were defined in this study.

The spatial distributions and concentrations of different HMs are illustrated in Figure 1 and Figure 2. The total concentration of HMs in the national agricultural soil ranged from 184.30 to 277.25 mg/kg. The considerably high HMs concentrations of 277.25 and 262.68 mg/kg in agricultural soil were observed in the Central and Southwest regions, respectively. The concentrations of HMs in other regions followed the descending order of South Coast (249.50 mg/kg), Central Coast (242.05 mg/kg), Northwest (240.75 mg/kg), Beijing-Tianjin (214.61 mg/kg), Northeast (200.95 mg/kg) and North (184.30 mg/kg). In addition, large variances in the concentrations of individual HMs were noted. Cd was the primary polluting element in agricultural soil, with concentrations ranging from 0.23 to 1.47 mg/kg. The higher concentrations of Cd were found in the Southwest (1.47 mg/kg) and Northeast (1.41 mg/kg) regions, exceeding the risk control standard for soil contamination of agricultural land. At the same time, Qin et al. [21] reported that the average concentration of Cd in agricultural soil in Yunnan Province was 1.19 mg/kg, and the concentration of Cd significantly exceeded the risk control standard for soil contamination of agricultural land.

The concentration of Zn was the highest, with a mean concentration of 93.06 ± 17.83 mg/kg, following the descending order of Central (117.30 mg/kg), South Coast (115.15 mg/kg), Central Coast (104.69 mg/kg), Southwest (100.59 mg/kg), Northwest (83.52 mg/kg), Beijing-Tianjin (82.61 mg/kg), Northeast (74.93 mg/kg), and North (65.72 mg/kg). The relatively higher concentrations of Zn were observed in the Hunan and Guizhou Provinces. The distribution of As was similar to that of Zn and was also highest in the Central (16.08 mg/kg) region, followed by Southwest (13.71 mg/kg) and Northwest (11.18 mg/kg) regions. The concentrations of As were generally lower for the remaining regions and less than 10.00 mg/kg. The distribution of Cr was relatively uniform across the country, with a mean concentration of 61.88 mg/kg. The highest level was observed in the Northwest (80.51 mg/kg) region, while the concentrations in other regions ranged from 39.89 to 68.39 mg/kg. The concentration of Ni was 34.17 ± 5.88 mg/kg, and the highest concentration of Ni was observed in the Southwest (46.95 mg/kg) region. In contrast with the national standards, the national Ni excess standard rate was 12.5% (Gansu, Anhui, Yunnan and Guizhou), indicating a relatively high level of Ni pollution in the soil. The highest concentration of Pb was observed in the South Coast (59.27 mg/kg) region, followed by the Central (39.92 mg/kg) and Southwest (37.90 mg/kg) regions. The concentrations of Pb were generally lower for the remaining regions (21.39–30.13 mg/kg).

Previous findings of higher Pb concentrations in Central China, specifically in Daye City, Hubei Province, were confirmed in this study. This region is known for its rich deposits of both ferrous and non-ferrous minerals [22], which may contribute to elevated Pb levels in soils. In this study, higher Zn concentrations were observed in Hunan and Guizhou Provinces, which can be attributed to abundant mineral resources, soil pollution associated with mining activities, and emissions from related industries involving potentially toxic elements [23,24]. In several provinces, soil Ni concentrations were found to exceed national standards, with Yunnan Province identified as the most affected. This pattern may be explained by agricultural activities conducted in carbonate parent material regions, where changes in soil physicochemical properties and structure have facilitated the mobilization of certain HMs. In addition, karst landforms are most extensively developed and widely distributed in Southwest China [25]. The findings indicate that hotspot cities for HMs in urban soils are primarily located in Southwest and Central China. These regions should therefore be prioritized for the implementation of HMs contamination control measures. Furthermore, higher contamination levels were observed in Southern China compared with Northern China, which may be attributed to elevated geochemical background levels and a prolonged history of industrial activity in these regions [26]. In addition, substantial contributions to HMs pollution may have resulted from urban population growth and the expansion of industrial activities, particularly within manufacturing sectors. In summary, relatively severe HMs contamination was identified in Central and Southwest China.

### 3.2. The Concentration of HMs in Rice and Wheat

The BCF of As in both rice and wheat was similar, and there were few BCF differences between the two crops, with BCF values around 0.0030 for As. The BCFs of Cr and Pb in rice and wheat were small, with BCF < 0.0050 for Cr and Pb, indicating a weak ability to enrich HMs. However, the BCFs of the other three HMs differed significantly between the two crops, indicating variations in the degree of enrichment. Notably, Pb had the lowest BCF value of 0.0014 in rice, suggesting that it was the most challenging metal to migrate from soil to rice. On the other hand, Cd and Zn exhibited the highest BCF values in rice, with BCF values of 0.0489 for Cd and 0.1619 for Zn. In wheat, the highest BCF values were observed for Cd (0.0414) and Zn (0.2345). Both crops demonstrated a strong enrichment effect for Cd and Zn, with Zn showing higher BCF values in both rice and wheat. These findings align with previous studies showing that Zn had the highest BCF value in wheat [27,28].

**Figure 3:**
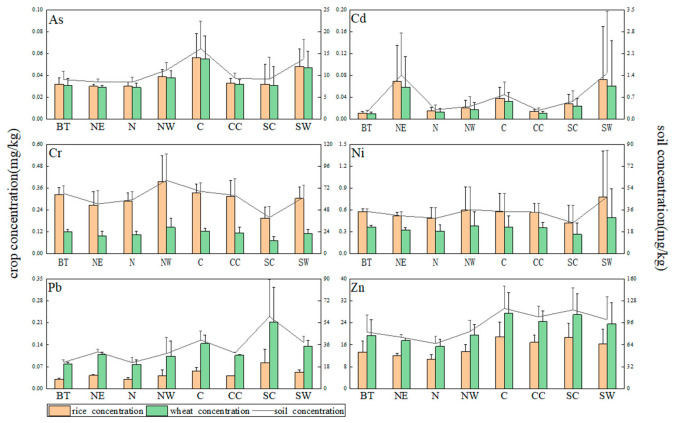
The estimated concentrations of HMs in crops in different regions.

Figure 3 presents the concentrations of HMs in wheat and rice across eight regions, calculated using the soil HMs occurrence data and the BCF values mentioned earlier. The total concentration of HMs in rice was 16.06 ± 2.91 mg/kg and in wheat was 22.48 ± 4.22 mg/kg. Compared with the total concentration of HMs in rice, the total concentration in wheat was relatively high, so more attention should be paid to wheat. For As, the concentration in rice ranged from 0.03 mg/kg to 0.06 mg/kg, with an average concentration of 0.04 ± 0.01 mg/kg. In wheat, the concentration was 0.04 ± 0.01 mg/kg, ranging from 0.03 mg/kg to 0.06 mg/kg in the eight regions. The limit of As in the national standard is 0.50 mg/kg, and the mean concentration of As in crops was within the range. It was noteworthy that the HMs concentrations of rice and wheat in the Central region differed significantly from the average concentration, suggesting a potentially higher risk associated with As in this region. The estimated concentration of As in rice in this study was similar to the estimated concentration of As in other studies (0.06 mg/kg) [29].

For Cd, concentrations in rice ranged from 0.01 to 0.07 mg/kg, with an average of 0.03 ± 0.02 mg/kg. In wheat, an average concentration of 0.03 ± 0.02 mg/kg was observed, with values ranging from 0.01 to 0.06 mg/kg across the eight regions. The national standard limit for Cd in cereals is 0.40 mg/kg, and the mean concentration in crops in this study was found to be within this threshold. For Cr, the concentration in rice was 0.30 ± 0.05 mg/kg, ranging from 0.20 to 0.40 mg/kg across the eight regions. In wheat, a lower concentration of 0.11 ± 0.02 mg/kg was observed, with a range of 0.07 to 0.15 mg/kg, values comparable to those reported in a previous study [30]. The national standard limit for Cr in cereals is 1.00 mg/kg, and the mean concentration in crops was within this range. A concentration of 0.48 ± 0.20 mg/kg in rice was reported in another study [31], which was comparable to the estimated value in this study (0.40 ± 0.20 mg/kg). For Ni, the concentration in rice was 0.57 ± 0.10 mg/kg, ranging from 0.42 to 0.78 mg/kg across the eight regions. A value of 0.52 ± 0.09 mg/kg was reported previously [32], which was consistent with the estimate obtained in this study. In wheat, the concentration was 0.36 ± 0.06 mg/kg, with a range of 0.27 to 0.49 mg/kg. The national standard limit for Ni in cereals is 1.00 mg/kg, and the mean concentrations in both wheat and rice were within this threshold; however, relatively high Ni concentrations observed in crops from Southwest China indicate potential risks to human health, and strengthened management in this region is warranted. For Pb, the concentration in rice was 0.05 ± 0.02 mg/kg, ranging from 0.03 to 0.08 mg/kg across the eight regions, which is consistent with previously reported values (0.05 mg/kg) [33]. In wheat, the concentration was 0.12 ± 0.04 mg/kg, with a range of 0.08 to 0.21 mg/kg. The national standard limit for Pb in crops is 0.20 mg/kg. Mean Pb concentrations in rice and wheat were within this limit; however, HMs concentrations in wheat from the South Coast were found to exceed the standard, indicating elevated risk in this area and the need for strengthened management. In addition, the concentration of Zn in rice was 15.07 ± 2.89 mg/kg in eight regions, with a range of 10.64 mg/kg to 18.99 mg/kg. In wheat, the concentration was 21.82 ± 4.18 mg/kg, ranging from 15.41 mg/kg to 27.51 mg/kg, which was similar to the concentration of Zn in rice (20.74 ± 3.98 mg/kg) found by Tong et al. [34]. The national standard limit for Zn in crops is 50.00 mg/kg, and the mean concentrations calculated in this study were within this range.

The concentrations of As, Cd, Cr and Pb in wheat were lower than those in rice, suggesting that the bioaccumulation of HMs in crops is related to crop species. In addition, the concentration of Pb in wheat in the South Coast region exceeded the standard, while that in soil did not. This cannot be attributed to a high BCF, as the BCF values of Pb in both rice (0.0014) and wheat (0.0036) are very low, indicating a limited transfer capacity from soil to crops. Pb accumulation in wheat in this region is more likely associated with other factors, such as atmospheric deposition, irrigation water inputs, or variations in soil physicochemical properties that affect Pb availability. The environmental supervision agency should prioritize this region and implement effective strategies to reduce the risk [35,36]. Regions such as the Southwest, Central, and Northeast show higher concentrations of HMs in both rice and wheat, as depicted in Figure 3. The accumulation of HMs in the environment, coupled with their uptake by crops, can have detrimental effects on human health [37].

### 3.3. The EDI of HMs from Interregional Trade

**Figure 4:**
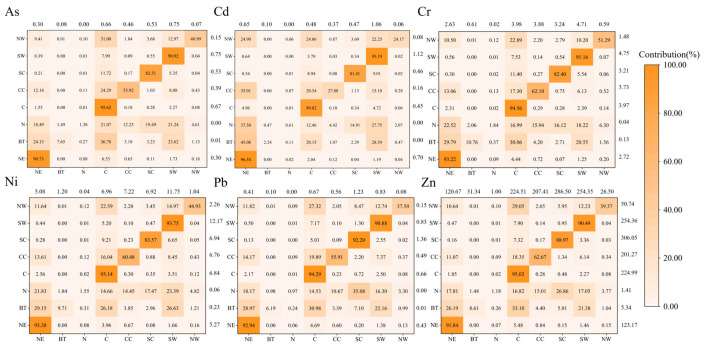
The dietary exposure of HMs in rice. (The value of each cell represents the intake contribution from other regions to the region labeled on the left. The value in each panel at the top is the EDI of HMs (μg/kg/day) considering food trade, while that on the right represents the EDI without considering food trade.).

**Figure 5:**
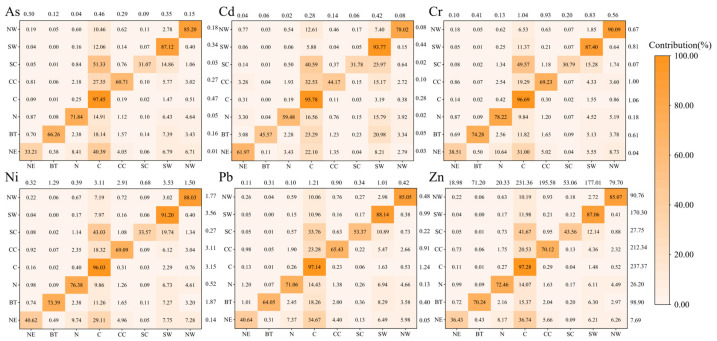
The dietary exposure of HMs in wheat. (The value of each cell represents the intake contribution from other regions to the region labeled on the left. The value in each panel at the top is the EDI of HMs (μg/kg/day) considering food trade, while that on the right represents the EDI without considering food trade.).

Dietary intake of HMs in each region may have come from other regions; therefore, the trade quantity of rice and wheat and the relative contribution of daily HMs intake in the local population needed to be considered [38]. The dietary exposures of HMs in rice and wheat are illustrated in Figure 4 and Figure 5. In most regions, more than 60% of EDI came from locally produced crops, especially for rice, where the proportion reached over 80% in the Northeast, Central, South Coast and Southwest regions. For instance, the dietary exposure of Cd in the Northeast region was 0.70 μg/kg/day, with 96.54% coming from locally produced rice, while the remaining portion was imported from other regions. In regions with significant local contributions, the impact of trade on HMs intake was minimal. However, in regions such as Beijing-Tianjin, North and Northwest, there were significant changes in HMs intake when considering the influence of trade. For instance, in Beijing-Tianjin, the intake of As increased from 0.01 μg/kg/day before trade to 0.08 μg/kg/day, while in the Northwest region, the intake decreased from 0.15 μg/kg/day to 0.07 μg/kg/day. Similar trends were observed for the intakes of five other metals. This was closely related to the main food in Beijing-Tianjin being rice, while in the Northwest, it is mainly wheat-based food such as noodles [39]. In addition, considering the influence of trade, the exposure to HMs by rice consumption in Beijing-Tianjin mainly came from the Northeast, Central and Southwest regions, with their contributions to the exposure being 84.53% for As, 93.82% for Cd, 80.40% for Cr, 81.96% for Ni, 82.11% for Pb, and 80.67% for Zn, respectively (Figure 4) [40]. Compared with the HMs exposure in Beijing-Tianjin, a portion of the HMs exposure in the North region also came from the South Coast region. The contributions from the four regions were 80.29% for As, 92.42% for Cd, 73.85% for Cr, 77.35% for Ni, 84.08% for Pb, and 78.54% for Zn, respectively. Overall, interregional trade was associated with both increases and decreases in HMs exposure across regions, indicating heterogeneous impacts across different regional trade patterns.

Furthermore, for the exposure by wheat consumption, 50.00%~60.00% of the EDI of HMs (except Cd) in the Northeast and South Coast regions came from food trade, especially from the Central region. The EDI of As from the Central region accounted for 40.39% in the Northeast region and 51.33% in the South Coast region, respectively. Similar patterns were observed for the other four HMs (Cr, Ni, Pb, Zn). Consumption of wheat in the Central region was the primary source of HMs exposure in other regions, followed by the Southwest region (Figure 5). However, the regional contribution of Cd daily intake resulting from the impact of food trade exhibited distinctive differences from other HMs. In the Northeast, the EDI of Cd mainly came from local production, accounting for 61.97%. The results indicated that the Central and Southwest were the primary exporters of HMs to other wheat-trading regions. This may be due to the presence of some major crop producers in the Central and Southwest regions [41]. Hence, the effects of trade on HMs concentrations in crops varied across different regions.

The interregional food trade had an impact on dietary exposure to HMs in rice and wheat. Specifically, in rice, the EDI of six HMs in the Northeast, Southwest and Northwest regions, As, Cr, Ni, Pb and Zn in the North region, As and Zn in the Central region, and Ni, Pb and Zn in the South Coast region were reduced because of food trade (Figure 4). Conversely, in other regions, trade led to an increase in EDI of HMs. Additionally, Figure 5 illustrates the impact of trade on HMs in wheat. The EDI of HMs was increased due to the interregional food trade, with the largest impact on As (62.70%), Cd (61.84%) and Cr (63.04%) in the South Coast region and As (63.09%) in the Northeast region. Conversely, interregional food trade led to a reduced EDI of HMs, with the greatest effect on Cr (31.92%) and Ni (31.10%) in Beijing-Tianjin. Similar results have been reported; food trade could increase the MeHg exposure through China [42], supporting that the interregional food trade had an influence on the EDI of HMs. The international food trade led to domestic environmental problems, and an analysis of international trade was needed to ensure global food security and environmental sustainability [43].

The EDI of HMs was influenced by both locally produced crops and interregional food trade. In regions with high concentrations of HMs, the EDI of HMs by the local population can be reduced by importing low-contamination crops or exporting high-concentration crops. Similar patterns have been observed in studies on other contaminants, such as PCB-153 in fish, where exporting fish from highly contaminated regions to less contaminated regions resulted in higher EDI of PCB-153 in the latter ones [44]. Consequently, it was suggested that interregional food trade cooperation be optimized in order to reduce the EDI across diverse regions.

### 3.4. Health Risk of HMs Associated with the Consumption of Rice and Wheat

**Figure 6:**
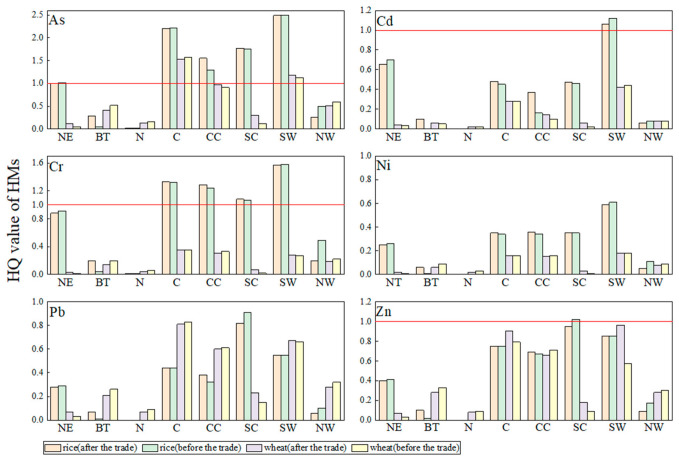
The estimated HQ values of HMs through rice and wheat in different regions (The red line indicates HQ > 1).

The HQ values for HMs in rice and wheat were calculated (illustrated in Figure 6) to evaluate the potential health risks of HMs exposure through diet. In wheat, only the HQ of As in the Central and Southwest regions was greater than 1.0, while the HQs caused by all HMs in the remaining regions were less than 1.00 within the range of 0.01–0.97. In rice, there were four HMs with HQ values greater than 1.00. The regions of HQ > 1.00 were as follows: As in the Northeast region, As and Cr in the Central and Central Coast regions, As, Cr and Zn in the South Coast region, and As, Cd and Cr in the Southwest region. The HQs in other regions were less than 1.00 within the range of 0.00–0.95. The results showed that As and Cr were the predominant HMs in rice and presented potential health risks. This finding aligned with a previous study [45]; therefore, it was crucial to focus more on managing HMs and strengthening control measures in order to enhance crop productivity. In addition, the contribution of six metals to health risk was calculated (Figure S1); the same conclusion was made that As and Cr had a relatively high contribution to risk and are more harmful to human health [46]. Moreover, in the Northeast, South Coast, Central, Central Coast and Southwest regions, the contribution of rice consumption to health risks was greater than that in the Beijing-Tianjin, North and Northwest regions, which might be attributed to variations in dietary habits within these regions [47].

Although the HQ values were less than 1.00 in most regions, the health risks of HMs were influenced by the interregional food trade. In wheat, the HQ values for Cd in Central Coast and the HQ values for As in Beijing-Tianjin were 0.16 and 0.04, respectively, before considering the impact of trade. However, after being affected by trade, the HQ values increased to 0.37 and 0.28, respectively. It was suggested that the influence of food trade can alter the health risks associated with HMs diets in each region. Liu et al. reported that up to 64% of MeHg exposure by consumption of fish and rice could be transferred to Beijing due to the food trade [48]. Furthermore, considering the rice trade, the HQ values of Pb decreased by 45.17% (from 0.10 to 0.06) and 9.66% (from 0.91 to 0.82) in the Northwest and South Coast regions, respectively. In these two regions, the Pb levels were relatively higher, and they had an export of high-level crops to other regions with lower Pb concentration. Similarly, the HQ value of Zn in the South Coast region was decreased from 1.02 to 0.95 because of the consumption of rice from regions with a lower Zn concentration. On the other hand, the HQ values of Cd via wheat in the Central Coast and North regions increased by 56.83% and 27.30%, respectively, due to the consumption of wheat imported from regions with high Cd concentrations. Studies have shown that, through food trade, crop consumption changes, thus affecting the risk to human health [49].

These findings suggested that interregional food trade could significantly impact the health risks caused by HMs contamination of crops. The direction and extent of trade influenced the HQ values in different regions, highlighting the importance of considering trade patterns and their effects on human health risk assessments. Indeed, the present study underscored the importance of considering the impact of food trade on HMs contamination and subsequent health risks. Through food trade, the HMs exposure caused by consuming crops grown on contaminated soil has been observed to pose potential risks to the population in China. The need for comprehensive monitoring, regulation, and management of food trade to ensure the safety and well-being of the population in terms of HMs exposure [50] is highlighted.

## 4. Conclusions

This study evaluated the influence of interregional food trade on the HMs exposures and the subsequent health risks posed by soil HMs pollution in China. The findings revealed regional variations in the EDI of HMs, with the highest levels investigated in the Southwest and Central regions, while the lowest ones were in Beijing-Tianjin, Northwest and North regions. Furthermore, the study highlighted the influence of interregional food trade on dietary-induced HMs exposure and the corresponding risks in different regions. Overall, the HMs exposure by crop consumption was reduced due to the interregional trade; however, an increase in exposure to Cd could not be ignored. The health risks of As, Cd and Cr are concerning. The export of food from regions with higher HMs concentrations could increase dietary exposure in regions with low HMs concentrations, emphasizing the need to examine the impact of pollutant flows from food trade on population health risks.

## Figures and Tables

**Table 1:** The eight regions of China.

The Name of Region	Abbreviation	Provinces Included in the Region
Beijing-Tianjin	BT	Beijing, Tianjin
Northeast	NE	Jilin, Heilongjiang, Liaoning
North	N	Shanxi, Hebei, Shandong
Northwest	NW	Ningxia, Shaanxi, Xinjiang, Gansu, Qinghai, Inner Mongolia
Central	C	Hubei, Hunan, Henan, Anhui, Jiangxi
Central Coast	CC	Shanghai, Jiangsu, Zhejiang
South Coast	SC	Fujian, Guangdong, Hainan, Hong Kong
Southwest	SW	Chongqing, Sichuan, Guangxi, Guizhou, Yunnan, Tibet

**Table 2:** The BCFs of selected HMs adopted in this study.

	As	Cd	Cr	Ni	Pb	Zn
Rice	0.0035	0.0489	0.0049	0.0166	0.0014	0.1619
Wheat	0.0034	0.0414	0.0018	0.0105	0.0036	0.2345

## Data Availability

The data are contained within the article. Additional data are available upon request from the corresponding authors.

## Supplementary Materials

The following supporting information can be downloaded at: https://www.mdpi.com/article/10.3390/toxics14060474/s1, Figure S1: The contribution of HMs to human exposure risk before and after rice and wheat trade in eight regions; Table S1: More detailed information regarding BCF [27,51,52,53,54,55,56,57,58,59,60,61,62,63,64,65,66,67]; Table S2: Rice consumption contributed by different regions; Table S3: Wheat consumption contributed by different regions.
