# Supplementary material for: Evaluating the Influence of Food Trade on Human Exposure to Heavy Metals via Crops in China
Source: Toxics. 2026 May 28;14(6):474. doi: 10.3390/toxics14060474 (PMC13306763; doi:10.3390/toxics14060474)
Supplement: Supplementary file 1 [file toxics-14-00474-s001.zip › toxics-4316389-supplementary.pdf]

Supplementary materials

Evaluating the Influence of Food Trade on Human Exposure to Heavy Metals via Crops in China

Bo Tang [1,2], Xuhua Miao [3,4,*], Jianyuan Ma [3,4], Wenxiu Liu [1,*], Qingbao Gu [1] and Fujun Ma [1]

[1] *State Key Laboratory of Environmental Criteria and Risk Assessment, Chinese Research Academy of Environmental Sciences, Beijing 100012, China;*

[2] *School of Chemical& Environmental Engineering, China University of Mining & Technology-Beijing, Beijing 100083, China;*

[3] *Gansu Academy of Eco-environmental Sciences, Lanzhou 730020, China;*

[4] *Gansu Engineering Research Center of Soil Environmental Protection and Pollution Prevention, Lanzhou 730020, China*

The following are included as supporting information for this paper

Number of pages: 9

Number of figures: 1

Number of tables: 3

*Corresponding author

E-mail address: 13993181102@163.com (Xu-hua Miao); Liuwx@craes.org.cn(Wen-xiu Liu)

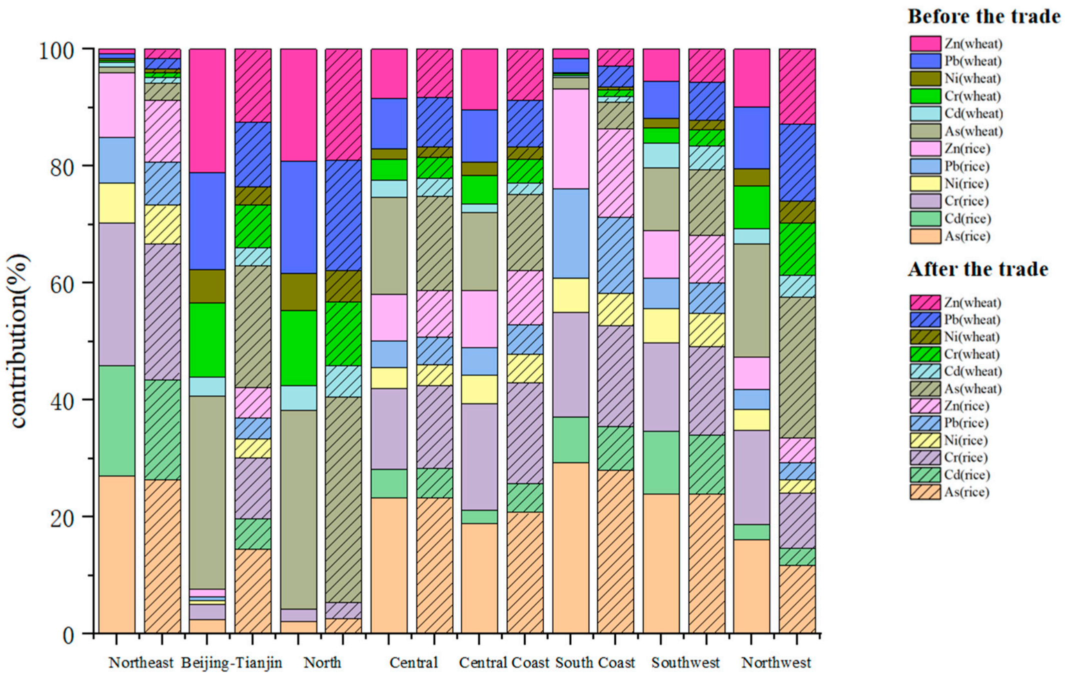

Figure S1. The contribution of HMs to human exposure risk before and after rice and wheat trade in eight regions.

Table S1. More detailed information regarding BCF

Crops/HMs	As	Cd	Cr	Ni	Pb	Zn	
rice			0.005		0.002		(Li et al., 2022)
rice		0.0163			0.001		(Chen et al., 2021)
rice		0.0043	0.0006			0.0329	(Liao et al., 2013)
rice	0.001	0.056		0.002	0.002	0.284	(Jiang et al., 2020)
rice	0.0072	0.139	0.005	0.0123	0.0011	0.138	(Fan et al., 2018)
rice	0.0066	0.0379	0.0035	0.0157	0.0002	0.1474	(Xu, 2021)
rice	0.0006	0.001	0.0001	0.0008	0.0002	0.03	(Peng, 2020)
rice	0.002	0.088	0.014	0.005		0.13	(Liu et al., 2006)
rice			0.009	0.064	0.003	0.34	(Wang et al., 2016)
rice			0.0035		0.0019	0.1548	(Yin et al., 2018)
rice			0.0032		0.0014	0.2	(Wu, 2020)
Mean (rice)	0.0035	0.0489	0.0049	0.0166	0.0014	0.1619	
wheat	0.001	0.0163	0.0015			0.0791	(Liao et al., 2013)
wheat	0.003						(Huang et al., 2008)
wheat	0.001						(Gao et al., 2004)
wheat	0.009				0.000542	0.054	(Wang et al., 2017)
wheat		0.028	0.005	0.021	0.002	0.318	(Teng et al., 2022)
wheat	0.003		0.0004	0.00004	0.002	0.446	(Cai & Song, 2019)
wheat					0.0087	0.32	(Wu, 2020)

wheat		0.08			0.005	0.19	(Zhu et al., 2017)
wheat			0.0002				(Sun et al., 2019)
Mean (wheat)	0.0034	0.0414	0.0018	0.0105	0.0036	0.2345	

Table S2. Rice consumption contributed by different regions

		rice trade consumption (g/kg/d)							
		Receptor regions							
		Northeast	Beijing-Tianjin	North	Central	Central Coast	South Coast	Southwest	Northwest
Source regions	Beijing-Tianjin	3.30E-04	2.02E-01	1.12E-03	1.09E-04	4.55E-04	3.14E-05	3.32E-05	1.77E-04
	Northeast	9.14E+00	6.77E-01	1.47E-02	3.43E-01	1.89E+00	3.69E-02	9.85E-02	2.32E-01
	North	7.87E-03	7.79E-03	1.12E-03	3.18E-03	1.74E-02	1.97E-03	1.52E-03	2.54E-03
	Northwest	1.35E-02	2.41E-02	2.80E-03	1.38E-02	5.16E-02	5.34E-03	8.28E-03	7.72E-01
	Central	3.48E-01	5.46E-01	8.90E-03	1.12E+01	2.00E+00	1.10E+00	1.06E+00	4.05E-01

Central Coast	6.01E-02	8.14E-02	8.90E-03	3.70E-02	7.67E+00	2.81E-02	2.09E-02	4.14E-02
South Coast	1.00E-02	8.43E-02	1.45E-02	5.74E-02	1.49E-01	1.37E+01	1.29E-01	8.46E-02
Southwest	1.08E-01	4.11E-01	1.05E-02	3.13E-01	7.82E-01	5.91E-01	1.41E+01	1.99E-01

Table S3. Wheat consumption contributed by different regions

		wheat trade consumption (g/kg/d)							
		Receptor regions							
		Northeast	Beijing-Tianjin	North	Central	Central Coast	South Coast	Southwest	Northwest
Source regions	Beijing-Tianjin	4.23E-03	2.58E+00	9.35E-04	1.40E-03	5.82E-03	4.02E-04	4.24E-04	2.27E-03
	Northeast	3.94E-01	2.91E-02	1.15E-02	1.48E-02	8.15E-02	1.59E-03	4.24E-03	1.00E-02
	North	1.01E-01	9.96E-02	9.56E-01	4.07E-02	2.22E-01	2.52E-02	1.94E-02	3.25E-02
	Northwest	6.07E-02	1.08E-01	4.66E-02	6.18E-02	2.32E-01	2.40E-02	3.71E-02	3.46E+00
	Central	2.54E-01	3.98E-01	1.04E-01	8.18E+00	1.46E+00	8.04E-01	7.71E-01	2.95E-01
	Central Coast	4.38E-02	5.93E-02	1.35E-02	2.69E-02	5.59E+00	2.05E-02	1.52E-02	3.02E-02

South Coast	6.26E-04	5.28E-03	1.26E-03	3.59E-03	9.35E-03	8.56E-01	8.09E-03	5.30E-03
Southwest	5.00E-02	1.90E-01	5.26E-02	1.45E-01	3.62E-01	2.73E-01	6.53E+00	9.20E-02

References

27 Cai, K.; Song, Z.F. Bioconcentration, Potential Health Risks, and a Receptor Prediction Model of Metal(loid)s in a Particular Agro-Ecological Area. *Appl. Sci.* **2019**, *9*, 1902. https://doi.org/10.3390/app9091902.

51. Chen, R.; Wang, Q.; Lv, J.G.; Wang, Z.S.; Gao, T. Multivariate correlation analysis of bio-accumulation with soil properties and potential health risks of cadmium and lead in rice seeds and cabbage in pollution zones, China. *Environ. Geochem. Health* **2021**, *43*, 3485–3503.
52. Fan, R.W.; Wang, S.X.; Wang, J.X.; Zhang, Z.N.; Gu, H.D. Distribution Characteristics and Dietary Exposure Risk Assessment of Selenium, Arsenic and Heavy Metals in Rice in Suzhou Region. *Adm. Tech. Environ. Monit.* **2018**, *30*, 37–41.
53. Gao, M.; Wei, Z.F.; Che, F.C.; Ci, E.; Xie, D.T. Effect of slog containing silicon on nutrient absorption and yield of rice. *Ecol. Environ.* **2004**, *13*, 587–591.
54. Huang, M.L.; Zhou, S.L.; Sun, B.; Zhao, Q.G. Heavy metals in wheat grain: Assessment of potential health risk for inhabit-ants in Kunshan, China. *Sci. Total Environ.* **2008**, *405*, 54–61.
55. Jiang, F.; Ren, B.Z.; Hursthouse, A.; Deng, R.J. Evaluating health risk indicators for PTE exposure in the food chain: Evidence from a thallium mine area. *Environ. Sci. Pollut. Res.* **2020**, *27*, 23686–23694.
56. Li, D.S.; Zhang, Q.H.; Sun, D.L.; Yang, C.L.; Luo, G.F. Accumulation and risk assessment of heavy metals in rice: A case study for five areas of Guizhou Province, China. *Environ. Sci. Pollut. Res.* **2022**, *29*, 84113–84124.
57. Liao, Q.L.; Liu, C.; Cai, Y.M.; Zhu, B.W.; Wang, C.; Hua, M.; Jin, Y. A preliminary study of element bioconcentration factors within milled rice and wheatmeal in some typical areas of Jiangsu Province. *Geol. China* **2013**, *40*, 331–340.
58. Liu, H.L.; Li, Y.H.; Li, L.Q.; Jin, L.; Pan, G.X. Pollution and risk evaluation of heavy metals in soil and agro-products from an area in the Tai hu Lake region. *J. Saf. Environ.* **2006**, (05): 60–63.
59. Peng, M. Heavy Metals in Soil-Crop System from Typical High Geological Background Areas, Southwest China: Transfer Characteristics and Controlling Factors. Ph.D. Thesis, China University of Geosciences, Beijing, China, 2020.
60. Sun, Z.Q.; Li, H.L.; Yu, X.Y.; Qiu, Y.H.; Han, X.M.; Li, S.W.; Ma, Y.B. Bioconcentration Ability of Chromium in Soil by Different Crops. *J. Univ. Jinan* **2019**, *33*, 255–260+265.
61. Teng, Y.; Ke, Y.Y.; Zhou, Q.X.; Tao, R.Q.; Wang, Y.B. Derived regional soil-environmental quality criteria of metals based on Anhui soil-crop systems at the regulated level. *Sci. Total Environ.* **2022**, *825*, 154060.
62. Wang, S.Y.; Wu, W.Y.; Liu, F.; Liao, R.K.; Hu, Y.Q. Accumulation of heavy metals in soil-crop systems: A review for wheat and corn. *Environ. Sci. Pollut. Res.* **2017**, *24*, 15209–15225.
63. Wang, T.Y.; Zhou, G.H.; Sun, B.B.; He, L.; Zeng, D.M.; Chen, Y.D.; Ye, R. The Relationship between Heavy Metal Contents of Soils and Rice in Coastal Areas, Fujian Province. *Rock Miner. Anal.* **2016**, *35*, 295–301.
64. Wu, Q.M. Study on Risk Assessment and Safe Utilization Technology of Heavy Metal in Farmland of Typical Rapid Economic Development Area. Master's Thesis, Nanjing University of Information Science and Technology, Nanjing, China, 2020.
65. Xu, J.Y. Bioavailability of Heavy Metals in Paddy Soils. Master's Thesis, Hefei University of Technology, Hefei, China, 2021.
66. Yin, Y.M.; Zhao, W.T.; Huang, T.; Cheng, S.G.; Zhao, Z.L.; Yu, C.C. Distribution Characteristics and Health Risk Assess-ment of Heavy Metals in a Soil-Rice System in an E-waste Dismantling Area. *Environ. Sci.* **2018**, *39*, 916–926.
67. Zhu, H.; Wu, C.F.; Chen, Y. Concentrations of Heavy Metals in Wheat Grains and Their Potential Health Risk in the Central Region of Jiangsu. *Adm. Tech. Environ. Monit.* **2017**, *29*, 35–38+56.
